# Gastric Cancer: Current Status of Diagnosis and Treatment

**DOI:** 10.3390/cancers5010048

**Published:** 2013-01-16

**Authors:** Tsunehiro Takahashi, Yoshiro Saikawa, Yuko Kitagawa

**Affiliations:** Department of Surgery, School of Medicine, Keio University, Shinjuku-ku, Tokyo 1608582, Japan

**Keywords:** gastric cancer, surgery, chemotherapy, radiotherapy, molecular targeted therapy

## Abstract

Gastric cancer is the second leading cause of death from malignant disease worldwide and most frequently discovered in advanced stages. Because curative surgery is regarded as the only option for cure, early detection of resectable gastric cancer is extremely important for good patient outcomes. Therefore, noninvasive diagnostic modalities such as evolutionary endoscopy and positron emission tomography are utilized as screening tools for gastric cancer. To date, early gastric cancer is being treated using minimally invasive methods such as endoscopic treatment and laparoscopic surgery, while in advanced cancer it is necessary to consider multimodality treatment including chemotherapy, radiotherapy, and surgery. Because of the results of large clinical trials, surgery with extended lymphadenectomy could not be recommended as a standard therapy for advanced gastric cancer. Recent clinical trials had shown survival benefits of adjuvant chemotherapy after curative resection compared with surgery alone. In addition, recent advances of molecular targeted agents would play an important role as one of the modalities for advanced gastric cancer. In this review, we summarize the current status of diagnostic technology and treatment for gastric cancer.

## 1. Introduction

Gastric cancer is the second leading cause of death from malignant disease worldwide, with especially high mortality rates in East, South, and Central Asia; Central and Eastern Europe; and South America [1]. In 2008, there were approximately 989,000 new cases of gastric cancer and 738,000 deaths worldwide. Gastric cancers are most frequently discovered in advanced stages, except in East Asia, where screening programs have been established. The prognosis of advanced gastric cancer remains poor, and curative surgery is regarded as the only option for cure. Early detection of resectable gastric cancer is extremely important for good patient outcomes; therefore, technologically sophisticated screening programs are needed. In the near future, however, improving the prognosis of advanced gastric cancer is necessary, and this includes multimodality treatment using chemotherapy, radiotherapy, and surgery. In this review, we summarize the current status of diagnostic technology and treatment for gastric cancer.

## 2. Diagnosis

### 2.1. Upper Gastrointestinal Endoscopy

It cannot be overemphasized that upper gastrointestinal endoscopy is indispensable for the diagnosis of gastric cancer. Recent innovations in the techniques used during upper gastrointestinal endoscopy have contributed to finding increased numbers of early-stage gastric cancers. Chromoendoscopy using indigo dye spraying previously played an important role in identifying early lesions. Magnifying endoscopy with narrow-band imaging (NBI) has undergone technological improvements, and has been reported to be accurate and reliable for the diagnosis of early gastric cancer [2,3]. Magnifying endoscopy with NBI allows observation of the microvascular architecture of the mucosa and microsurface pattern of the lesion, and is useful for assessing the area of the lesion and the depth of tumor invasion. Future validation of the correlation of NBI images with pathological findings is expected. Furthermore, endocytoscopy, a technique allowing microscopic visualization of the mucosal surface, has been developed [4,5]. Endocytoscopic visualization of nuclei might allow pathological diagnosis without the need for biopsy. This novel technology may play a major role in the diagnosis of early gastrointestinal cancer in the near future. Another diagnostic innovation is virtual endoscopy, which uses multidetector-row computed tomography (CT) [6]. With further development, followed by studies evaluating its diagnostic accuracy, it is anticipated that this noninvasive diagnostic modality will be established as a screening tool for gastrointestinal disease.

### 2.2. ^18^F-Fluorodeoxyglucose (FDG)-Positron Emission Tomography (PET)

The outcomes of patients with advanced gastric cancer strongly depend on whether or not curative resection is performed. Metastases to the liver and lymph nodes may be associated with noncurative resection, and have become more accurately diagnosed because of recent advances in diagnostic imaging technologies such as CT and magnetic resonance imaging (MRI). There have also been several reports on the effectiveness of PET for staging gastric cancer [7,8]. The incidence of metastasis in bone is not as high in patients with gastric cancer, with a rate of 0.7–1.4% [9]. Bone scintigraphy is recognized as the most useful examination to detect bone metastasis. Early detection of bone metastasis is important in order to prevent pathologic fractures and differentiate from a diagnosis of postoperative metabolic bone disease. In addition to bone scintigraphy, FDG-PET/CT is also useful for early detection of the bone metastasis. FDG-PET/CT has proved especially beneficial for identifying metastases that could not be detected by conventional imaging such as enhanced CT. We have had patients with increasing levels of tumor markers found during follow up after curative surgery but with negative findings on conventional imaging. The addition of FDG-PET/CT might prove useful in identifying occult metastases in these patients, and facilitate treatment planning for both preoperative and postoperative cases of gastric cancer. A recent prospective study demonstrated that preoperative PET was useful for accurate staging and also for reducing the costs of patient care [10]. PET has also been reported to be useful for assessing response to chemotherapy; changes in the metabolism of glucose by tumor cells have been reported to appear early during chemotherapy [11]. When preoperative chemotherapy is administered, determining the best time for surgical intervention requires timely and accurate assessment of the effects of chemotherapy. Further clinical studies are needed to determine if PET is an accurate diagnostic modality that can provide survival benefits for patients with gastric cancer.

### 2.3. Staging Laparoscopy (SL)

Peritoneal dissemination, which has been difficult to identify using conventional diagnostic imaging techniques, is the most frequent noncurative manifestation of gastric cancer. In some patients, peritoneal dissemination cannot be found before surgery, and laparotomy becomes an exploratory procedure after dissemination is discovered. Staging laparoscopy (SL) is a minimally invasive, brief procedure that only requires a small incision. There have been many reports, dating from the 1990s, on the usefulness of SL for staging gastric cancer [12,13]. The advantages of SL include providing an accurate diagnosis of peritoneal dissemination and extraserosal invasion, and the ability to perform peritoneal lavage for cytology. In patients with advanced gastric cancer for whom imaging does not yield a diagnosis, peritoneal lavage cytology obtained before treatment can be very important for treatment planning. Peritoneal lavage cytology obtained by SL for evaluation of peritoneal dissemination is thought to be useful for assessing the effects of neoadjuvant chemotherapy. Negative findings on peritoneal lavage cytology can aid in the decision to perform resection after neoadjuvant chemotherapy. Gastric cancer patients with positive findings on peritoneal lavage cytology are considered to have stage IV disease, based on the seventh edition of the TNM classification for gastric cancer [14]. Carcinomatous peritonitis occurs at a high rate even without dissemination in patients who are found to have free intraperitoneal cancer cells on peritoneal lavage cytology [15,16]. Therefore, staging laparoscopy can be applied to patients with very advanced gastric cancer and potential peritoneal dissemination. SL is probably useful for patients with the following: (1) endoscopic or CT findings suggesting extraserosal invasion, (2) scirrhous gastric cancer, which tends to disseminate throughout the peritoneum, (3) findings suggesting peritoneal dissemination or a small amount of ascites, and (4) indications for neoadjuvant chemotherapy.

## 3. Treatment

### 3.1. Endoscopic Resection

Endoscopic mucosal resection (EMR) has dramatically changed the treatment of early gastric cancer [17]. EMR is a minimally invasive procedure that provides curative resection of the tumor via the lumen of the stomach. Because the volume of the stomach is maintained, the patient retains a good quality of life, and postgastrectomy complications due to gastric dysfunction and reduced gastric volume are prevented. EMR and endoscopic submucosal dissection (ESD) are indicated for patients in whom the probability of lymph node metastasis is low. According to the Japanese treatment guidelines for gastric cancer, EMR is a standard treatment for differentiated mucosal gastric cancer measuring less than 2 cm and without signs of ulceration [18]. En bloc resection is required to evaluate the surgical margin for confirmation of curative resection. ESD, which can be performed because of the development of the insulation-tipped diathermic knife (IT-knife) and the flex-knife for endoscopic procedures, can be used for en bloc resection of larger tumors [19,20]. Therefore, the indications for endoscopic treatment of patients with gastric cancer may be broadened. New indications for minimally invasive treatment will require evaluation before implementation.

### 3.2. Laparoscopic Surgery

Since the first laparoscopic cholecystectomy in the 1980s, laparoscopic surgery has been used for various applications, including gastrointestinal surgery [21,22,23]. Laparoscopic surgery is a minimally invasive procedure used in patients with gastric cancer who are not appropriate for endoscopic treatment [24]. The benefits of laparoscopic surgery include less postoperative pain, better cosmetic results, and early recovery. The technical issues of laparoscopic surgery for gastric cancer center on lymph node dissection and reconstruction. When laparoscopic gastrectomy was first introduced, D1 lymph node dissection was performed because of the technical difficulties involved in systemic lymph node dissection. However, advances in laparoscopic instrumentation, such as an ultrasonic coagulating shears to seal blood vessels, have made feasible extended lymph node dissection that includes suprapancreatic lymph nodes. The frequency of D2 lymph node dissection has gradually increased because of these advances [25]. Laparoscopy-assisted surgery has been widely performed in reconstruction in which the intestine is pulled out of the body through a small laparotomy wound. Reconstructive methods have included laparotomy, Billroth-I reconstruction, and Roux-en-Y reconstruction. Recently, pure laparoscopic surgery has also been used in which a series of procedures of lymphadenectomy, resection, and reconstruction is completely performed intraabdominally [26]. In addition, recent improvement in anastomosis devices and modifications in various anastomotic techniques have enabled esophagojejunostomy and anastomosis between the esophagus and remnant stomach. Laparoscopy-assisted proximal gastrectomy (LAPG) and laparoscopiy-assisted total gastrectomy (LATG) have begun to be performed. In particular, the introduction of circular stapler with transorally inserted anvil has enabled esophagojejunostomy and esophagus-remnant stomach anastomosis [27]. These procedures closely approach conventional anastomosis by laparotomy.

One study retrospectively analyzed safety and curative potential of laparoscopic surgery for gastric cancer [28]. The study involved 1,294 patients who had undergone laparoscopic gastrectomy between 1994 and 2003 in 16 surgical units (the Japanese Laparoscopic Surgery Study Group). Their short- and long-term outcomes were examined. For safety, the postoperative complications occurred in 14.8% of the patients, and the mortality rate associated with the operation was 0%. The 5-year disease-free survival rate was 99.8% for stage IA disease, 98.7% for stage IB disease, and 85.7% for stage II disease. These therapeutic outcomes were non-inferior to those of laparotomy in past reports. Table 1 summarizes the randomized controlled trials reported to date [29,30,31,32,33]. These reports showed safety and effectiveness of laparoscopic surgery. However, there are no results from large-scale randomized controlled trials. Thus, it cannot be said that a sufficient level of evidence is currently available. Clinical trials are underway to establish new evidence. The Japan Clinical Oncology Group (JCOG) conducted a phase II trial (JCOG 0703) in early cancer patients in whom endoscopic treatment was not indicated [34]. There were 176 enrolled patients. The primary endpoints were incidences of anastomotic leak and pancreatic fistula, which were 1.1% (two patients) each. This trial demonstrated the safety of laparoscopic-assisted distal gastrectomy (LADG) in early cancer patients. Thus, the randomized controlled clinical trial JCOG 0912 was begun to compare LADG and open distal gastrectomy (ODG). The Korean Laparoscopic Gastrointestinal Group completed patient recruitment for a randomized controlled trial (KLASS I) in early cancer patients and reported postoperative complications [35]. There were 342 enrolled patients (LADG: 179 patients, ODG: 161 patients). The incidence of postoperative complications was 10.5% and 14.7% in the LADG group and ODG group, respectively. The incidence of surgery-related deaths was 1.1% and 0%, respectively. The two groups had no significant difference in these incidences, and the safety of LADG was indicated. Presently, there is an ongoing examination of survival time.

**Table 1:** Randomized controlled trial of laparoscopic surgery.

Reference	Eligibility	n	Endpoints
Kitano, *et al*. (2002) [28]	T1	28	Operative findings, postoperative course and pulmonary function
Huscher, *et al*. (2005) [29]	TanyNanyM0	59	Operative findings, postoperative course, pathologic findings and overall survival
Lee, *et al*. (2005) [30]	T1	47	Operative findings, postoperative course, pathologic findings
Hayashi, *et al*. (2005) [31]	T1	28	Operative findings, postoperative course and enzyme immunoassays
Kim, *et al*. (2008) [32]	T1	164	Operative findings, postoperative course and QOL questionnaires

Surgery must show good short-term outcome and must be safe, without decreasing curative potential, which is the aim of cancer treatment. Ongoing clinical trials should elucidate long-term outcomes, including survival time. However, ongoing randomized controlled trials are limited to certain institutions and surgeons. Thus, it is difficult to use the results from these clinical trials to establish standard treatment. In addition, the quality of surgery must be maintained, and the standard treatment will likely be required to be performed by skilled surgeons with years of experience.

Robotic-assisted surgery has been developed recently for use in general and pediatric surgery, gynecology, and urology [36,37,38,39]. Although it has several disadvantages such as an absence of tactile feeling and expensive medical costs, robotic-assisted surgery may be used to develop minimally invasive surgical techniques that are a step ahead due to technological innovations. This new technology has resulted in a reduction in hand tremor and scale of hand movement of surgeons during laparoscopic surgery. In addition, the multi-articulated motion of robotic arms has led to more precise operations in gastric surgery. The usefulness of robotic-assisted surgery therefore needs to be validated in various fields in the near future.

### 3.3. Lymphadenectomy

In gastric cancer surgery, the approach to lymphadenectomy differs between European countries and East Asian countries, including Japan. There were two large-scale phase III trials in Europe: the MRC trial and a Dutch trial [40,41]. The results from these trials did not demonstrate the efficacy of D2 lymphadenectomy, and D1 lymphadenectomy is the standard lymphadenectomy. In Japan and Korea, there have not been any trials that showed the effectiveness of D2 lymphadenectomy compared with D1 lymphadenectomy. Since safety and effectiveness of dissection are high, there is not much significance in clinical trials that compare D1 and D2 lymphadenectomy. D2 lymphadenectomy is the standard procedure for gastric cancer cases in which curative resection is possible. In Japan, the randomized controlled trial JCOG 9501 was conducted in advanced gastric cancer patients to compare the clinical significance of D2 and D2 plus paraaortic lymphadenectomy (superextended lymphadenectomy, D3 lymphadenectomy) [42,43]. The subjects were patients in whom curative resection was possible and excluded patients with depths of tumor invasion of SS-SI or with CY0 type 4 gastric cancer. The group with D2 plus paraaortic lymphadenectomy had longer operative time and more blood loss than the group with D2. However, the incidence of postoperative complications did not differ significantly between the groups [42]. Thus, the safety of paraaortic lymphadenectomy was confirmed. The 5-year survival rate did not differ significantly between the D2 lymphadenectomy group and the D2 plus paraaortic lymphadenectomy group (70.3% and 69.2%, respectively) [43]. Paraaortic lymphadenectomy was not shown to have significance as prophylactic lymphadenectomy. Therefore, the conclusion was that superextended lymphadenectomy cannot be recommended in advanced gastric cancer patients. In the subset analysis, the prognosis was good when paraaortic lymphadenectomy was performed in a group with T2 (SS) and a group that was histologically negative for lymph node metastasis. Thus, paraaortic lymphadenectomy might be effective. It might be important to reexamine paraaortic lymphadenectomy in future studies with refined selection criteria of subjects.

### 3.4. Chemotherapy

**Figure 1:**
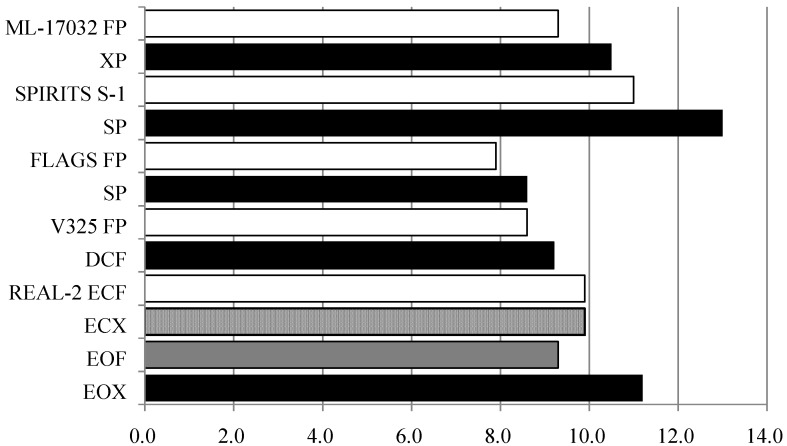
Median survival time of phase III trials of chemotherapy for unresectable gastric cancer were summarized.

Over 50 years have passed since 5-fluorouracil (5-FU) was developed, but it still plays a key role in the chemotherapeutic regimens for unresectable gastric cancer. The antitumor effects of 5-FU are enhanced when used in combination with cisplatin (CDDP) (FP therapy) due to biochemical modulation, and this therapy has yielded good therapeutic outcomes. FP therapy is one of the standard treatments that has been widely used and has been used as the reference arm in various clinical trials [44,45,46]. Oral 5-FU derivatives capecitabine and S-1 have been developed in recent years, and these drugs have been used instead of 5-FU in some clinical trials [44,45]. The ML-17032 trial was a controlled trial that compared FP therapy and a combination of capecitabine, a 5-FU prodrug, and CDDP (XP therapy) [44]. Progression-free survival (PFS) was 5.0 months in the FP group and 5.6 months in the XP group. Overall survival (OS) was 9.3 months and 10.5 months, respectively, indicating non-inferiority of the XP group. The SPIRITS trial compared S-1 monotherapy and S-1 plus CDDP (SP) therapy [47]. OS was 11.0 months in the S-1 monotherapy group and 13.0 months in the SP therapy group, indicating significantly higher OS in the SP group. In Japan, SP therapy has become the first choice for primary treatment. The FLAGS trial in the U.S. comparing of SP therapy over FP therapy, S-1 is a synthetic compound containing tegafur, gimeracil, and oteracil [45]. SP therapy has been shown to be safe with low hematological toxicity, low non-hematological toxicity, and low rate of treatment-related deaths. However, OS was 8.6 months in the SP group and 7.9 months in the FP group, not indicating superiority of the SP group. In the U.S., the V-325 trial compared FP therapy and the triple-drug therapy of docetaxel, cisplatin, and 5-FU (DCF). Time-to-progression (TTP) was 5.6 months in the DCF group and 3.7 months in the FP group, and OS was 9.2 months and 8.6 months, respectively [46]. There were significant differences in TTP and OS between the groups. However, there was high toxicity of DCF, including neutropenia and diarrhea. Thus, there are problems with DCF that prevent it from becoming the global standard treatment. In Europe, the efficacy of triple-drug therapies has been demonstrated. The REAL-2 trial had a 2 × 2 factorial design to compare four regimens [48]. One of the regimens was the conventional standard therapy of epirubicin, cisplatin, and fluorouracil (ECF therapy). In other regimens, fluorouracil was replaced by capecitabine (ECX therapy) in ECF therapy, cisplatin was replaced by oxaliplatin (EOF therapy), and both cisplatin and fluorouracil were replaced by oxaliplatin and capecitabine (EOX therapy). The primary endpoint was overall survival. Non-inferiority in overall survival was demonstrated for capecitabine- *vs*. 5-FU-based regimens (hazard ratio 0.86) and for oxaliplatin- *vs*. cisplatin-based regimens (hazard ratio 0.92). For the secondary endpoint, the overall survivals were compared among regimens. The overall survival was 9.9 months for the standard therapy of ECF, 9.9 months for ECX therapy, 9.3 months for EOF therapy, and 11.2 months for EOX therapy. For side effects of EOX therapy, there were low incidences of neutropenia and alopecia and high incidences of peripheral neuropathy and diarrhea. In Europe, because of its low toxicity and high efficacy, EOX therapy includes the oral drug capecitabine and oxaliplatin which does not require hydration has been recommended as one of the regimens. In the U.S., Europe, and East Asia, recommended regimens are being determined for primary treatment of unresectable advanced gastric cancer and Figure 1 summarizes the therapeutic outcomes of the clinical trials. In gastric cancer, new anticancer agents have not been developed after CPT-11 and taxanes. Since there are limited anticancer agents, combinations of anticancer agents are important for proceeding beyond secondary treatment. In addition, development of new anticancer agents will be essential.

### 3.5. Adjuvant Therapy

There are certain limitations when advanced gastric cancer is treated only with surgery. In the U.S., Europe, and Asia, there have been attempts to improve therapeutic outcomes using chemotherapy and chemoradiation therapy as neoadjuvant and adjuvant therapies. In the U.S., the INT-0116 trial examined the effectiveness of adjuvant chemoradiation therapy (CRT) using 5-FU/LV after curative resection (R0) in patients with stages IB-IV (M0) gastric cancer and esophagogastric junction cancer [49]. Overall survival (OS) was 27 months in the group that received surgery alone and 36 months in the group that received surgery and adjuvant CRT, indicating significantly longer OS in the latter group. Relapse-free survival was 19 months and 30 months, respectively, indicating significantly longer survival in the latter group. In the U.S., the results of this trial established chemoradiation therapy as the standard treatment for adjuvant therapy after curative resection. One limitation of this clinical trial was that D2 lymphadenectomy was performed in only 10% of the subjects. Thus, it cannot be said that the surgeries involved sufficient lymphadenectomies. The therapeutic outcomes of adjuvant CRT might be explained by radiation therapy compensating for the lack of local control using surgery. The ARTIST trial examined the addition of radiation therapy (RT) to the chemotherapy of capecitabine plus CDDP (XP) in adjunctive therapy after D2 lymphadenectomy [50]. Regarding safety, tolerability was high with low hematological and non-hematological toxicities. The primary endpoint of the 3-year disease-free survival rate was 74.2% in the XP group and 78.2% in the XP+RT group, indicating no significant difference. Thus, the addition of RT was not effective. Stratified analysis showed that the 3-year disease-free survival rate was 72.3% in the XP group and 77.5% in the XP+RT group in patients with histological lymph node metastasis. There was significant difference between the two groups. A trial is planned using appropriate patient selection and there is much expectation in the results of the trial.

In Europe, the MAGIC trial was conducted in patients with curatively resectable gastric cancer, esophagogastric junction cancer, and lower esophagus cancer [51]. It compared surgery alone and surgery plus perioperative chemotherapy, consisting of three preoperative and three postoperative cycles of epirubicin, CDDP, and 5-FU (ECF therapy). The primary endpoint of the 5-year survival rate was 36% in the perioperative chemotherapy group and 23% in the surgery-only group, indicating a higher survival rate in the perioperative chemotherapy group. The secondary endpoints of progression-free survival and down-staging rate were also significantly better in the perioperative chemotherapy group. The two groups had similar incidences of postoperative complications and mortality rates within 30 days of surgery. Although the completion rate of 3-course preoperative chemotherapy was high at 86%, only 42% of the patients completed postoperative ECF therapy. Improvement is needed before perioperative ECF therapy can be considered a standard treatment. The CRITICS trial is being conducted mainly in the Netherlands to compare two adjunctive therapies: perioperative ECF therapy and adjuvant CRT, as previously described as a standard treatment in the U.S [52]. The results of this study are expected to determine the direction of adjunctive therapy in Europe and the U.S.

Trials on adjuvant therapies were also conducted in East Asia where D2 lymphadenectomy is a standard procedure. In Japan, the ACTC-GC trial compared surgery alone and surgery plus adjuvant S-1 chemotherapy for curatively resectable advanced gastric cancer [53]. The primary endpoint of the 5-year survival rate was 71.7% in the surgery plus S-1 group and 61.1% in the surgery-only group, indicating a significant difference between the groups. The hazard ratio of the relapse risk was 0.653, and the relapse risk of 34.7% decreased using adjunctive therapy. In stratified analysis, a significant difference was seen in stage II patients but the additive effect tended to be low in stage III patients. However, in Japan, S-1 adjuvant therapy has been established as a standard treatment after curative resection for advanced gastric cancer. The CLASSIC trial was conducted in three countries—South Korea, China, and Taiwan—and examined the effectiveness of capecitabine plus oxaliplatin (XELOX) [54]. The primary endpoint of the 3-year disease-free survival (DFS) rate was 74% in the XELOX group and 59% in the surgery-only group. The hazard ratio was 0.56, and a significant difference was observed between the groups. Stratified analysis revealed a significant difference between the two groups in stage III patients, the type of patients with weak therapeutic effects in the ACTS-GC trial. Thus, XELOX used in combination with surgery has potential as adjunctive therapy for advanced stage gastric cancer. Since the concept of surgery differs by region of the world, consensus has not been reached regarding adjunctive therapy due to such differences. However, it will be essential to establish adjuvant therapy that patients can continue receiving while maintaining QOL without decreasing its therapeutic effects.

### 3.6. Radiation Therapy

Technological advancements in three dimensional conformal radiation therapy (3DCRT) and intensity modulated radiation therapy (IMRT) have enabled pinpoint treatment. These treatments have been performed as a part of the multidisciplinary therapy, mainly as adjunctive therapy. Radiation therapy is frequently used as palliative treatment and has been used in combination with chemotherapy. Since the late 1960s, several retrospective clinical trials have been conducted that compared CRT and chemotherapy (CT) alone or compared CRT and radiation therapy (RT) alone [55,56,57]. In 1968, Childs *et al*. reported significantly longer survival of 11.6 months in patients who underwent CRT compared with 5.7 months in patients who underwent RT alone [55]. Falkson *et al*. compared CT of 5-FU alone, RT, and CRT in a clinical trial. The response rate was good at 55% in patients with CRT, while it was 17% in patients with CT alone and 0% in patients with RT alone [51]. Subsequently, Klaassen *et al*. reported on a clinical trial of the Eastern Cooperative Oncology Group (ECOG), which compared 5-FU monotherapy and 5-FU plus RT for unresectable gastric cancer and pancreatic cancer [57]. The median survival was 9.3 months in patients with CT alone and 8.2 months in patients with CRT, indicating no significant difference. However, the number of subjects was small in these trials and the results did not demonstrate the effectiveness of CRT. There have been some reports on RT and CRT in Japan, but the number of subjects was small [58]. In addition, these treatments were often used as palliative treatment such as for pain, stenosis, and bleeding [59,60]. There have not been any recent reports on large-scale clinical trials regarding CRT for unresectable advanced gastric cancer. In recent years, the standard treatments of chemotherapy have been changing, including molecularly targeted drugs. New clinical trials are needed to reevaluate multidisciplinary treatment, including CRT, with appropriate patient selection.

### 3.7. Molecular Targeted Agents

In the chemotherapy for advanced cancer, it will be difficult to obtain marked improvements beyond presently achievable outcomes using only existing anticancer drugs. In the treatment of gastric cancer, there are high expectations for molecularly targeted agents which have played an important role in colorectal, breast, and lung cancer treatments. Trastuzumab is a humanized monoclonal antibody that selectively binds to the human epidermal growth factor receptor type 2 (HER2) and has improved the prognosis of HER2-postive breast cancer previously considered to have poor prognosis [61]. HER2 over-expression has been reported in 13–20% of gastric cancer cases [62,63]. There has been much expectation in the introduction of trastuzumab as a gastric cancer treatment. The ToGA study, a global clinical trial, examined the effectiveness of adding trastuzumab to chemotherapy (FP or XP therapy) for HER2-positive gastric cancer [64]. OS was 11.1 months in patients who received chemotherapy alone and 13.8 months in patients who received chemotherapy plus trastuzumab, indicating a significant difference. In particular, OS was 16.0 months when analysis was performed using only the strongly positive group [immunohistochemistry (ICH) 2+ and FISH positive, or ICH 3+]. These results helped establish chemotherapy plus trastuzumab as the standard treatment for HER2-positive gastric cancer just as in breast cancer.

Bevacizumab is an angiogenesis inhibitor that has played an important role in the treatment of colorectal cancer. The AVAGAST trial examined the effectiveness of bevacizumab addition to chemotherapy [65]. The response rate (RR) was significantly higher in patients who received bevacizumab plus chemotherapy compared with patients who received chemotherapy (XP or FP) alone (46% *vs*. 37%). The primary endpoint of OS tended to be higher in the combination therapy group but there was no significant difference (12.1 months *vs*. 10.1 months). Panitumumab and cetuximab are EGFR-inhibitors that have played an important role in the treatment of colorectal cancer. The REAL-3 trial was conducted in Europe to examine the effects of panitumumab addition to a standard therapy consisting of EOC [66]. The primary endpoint of OS was 8.8 months in the combination therapy group and 11.3 months in the EOC therapy group. OS was significantly shorter in the combination therapy group. However, it should be noted that the doses of chemotherapy were low in the combination therapy group. The EXPAND trial examined the effectiveness of cetuximab addition to chemotherapy. There were no significant differences in the primary endpoint of PFS, OS, or RR, indicating negative results [67]. Table 2 summarizes ongoing clinical trials of molecular targeted agents. Although the ToGA trial showed the effectiveness of trastuzumab, no other trials have produced positive results for gastric cancer. It will be desirable to find molecular markers for selection of patents in whom improvement of prognosis can be expected using molecular drugs.

**Table 2:** Ongoing phase III clinical trials of molecular targeted agents in gastric cancer.

Study	Line	Molecular targeted agent	Control arm	Primary endpoint
LOGiC	1st	Lapatinib	Capecitabine/Oxaliplatin	Overall survival
TYTAN	2nd	Lapatinib	Paclitaxel	Overall survival
RAINBOW	2nd	Ramuciruab	Paclitaxel	Overall survival
GRANITE-1	2nd/3rd	Everolimus	Placebo	Overall survival
GRANITE-2	2nd	Everolimus	Paclitaxel	Overall survival

## 4. Conclusions

The broad term “gastric cancer” includes varying stages of disease, but treatment plans differ greatly depending on the stage. Early gastric cancer is being treated using minimally invasive methods such as endoscopic treatment. For advanced cancer, if extended surgery is not indicated, multidisciplinary treatment, including chemotherapy, is used to improve treatment outcome beyond that attainable by surgery alone. Multidisciplinary treatment is being used in curatively resectable cases. There are many ongoing clinical trials that attempt to make breakthroughs, including in molecular drugs. There is much expectation that knowledge will be enhanced based on new scientific evidence, leading to advancement in gastric cancer diagnosis and treatment.
